# Progress and Challenges in Elimination of Vertical HIV Transmission

**DOI:** 10.1002/jia2.70131

**Published:** 2026-06-19

**Authors:** Lynne M. Mofenson, Jorge Pinto, Angela Mushavi

**Affiliations:** ^1^ Elizabeth Glaser Pediatric AIDS Foundation Washington, DC USA; ^2^ School of Medicine Federal University of Minas Gerais Belo Horizonte Brazil; ^3^ AIDS and TB Unit Ministry of Health Harare Zimbabwe

There has been tremendous progress in the prevention and treatment of vertical HIV transmission in children over the last three decades. In the early 1990s, vertical transmission from a mother living with HIV to her infant was approximately 15%–30% in high‐resource settings and 30%–45% in breastfeeding populations in lower‐resource settings, and almost 50% of children living with HIV died by the age of 2 years [1, 2, 3]. Research in maternal and paediatric HIV has been incremental over the years, with each study building on previous data, resulting in the current era of universal treatment with potent antiretroviral treatment (ART). With current regimens, vertical transmission rates from pregnant mothers living with HIV who achieve and maintain viral suppression are less than 1%–2%, and children with HIV are living into adolescence and young adulthood and having children of their own [4, 5, 6]. Thus, the tools to eliminate vertical transmission and end paediatric AIDS are available [7], and the goal of global elimination of new paediatric HIV acquisitions has been thought to be imminent [8].

However, while 84% of pregnant women living with HIV are currently receiving ART, overall coverage has been unchanged since 2019 [9]. Although globally there has been a 56% decrease in new vertical transmissions since 2010, more than one in every 10 HIV‐exposed infants acquired HIV in 2024. Almost half of the 120,000 new infant HIV acquisitions globally can be attributed to pregnant and breastfeeding mothers not knowing their HIV status and hence not receiving ART, and another quarter to new maternal acquisition of HIV during pregnancy or breastfeeding. While scale‐up of infant HIV diagnosis and treatment has improved over the years, the proportion of children undergoing early infant diagnostic testing was only 65% in 2024. Additionally, just over half of children living with HIV were receiving ART. This reflects a consistent gap between adults and children with HIV in achieving the UNAIDS 95‐95‐95 targets of knowing their HIV status, and being on treatment and virally suppressed [9, 10]. The stalling in prevention of paediatric HIV acquisition has several potential causes, including gaps in HIV testing for pregnant women; gaps in provision of and adherence to pre‐exposure HIV prophylaxis (PrEP) in women at risk of HIV; difficulties in adherence to ART and retention in care for women, particularly during the postpartum period; and limitations in formulations and availability of antiretroviral drugs for neonates.

Additionally, the global context for HIV prevention and care has dramatically changed since January 2025, with sudden and unexpected reductions in United States funding for the President's Emergency Plan for AIDS Relief (PEPFAR) and closure of the United States Agency for International Development. Although waivers for resumption of “urgent life‐saving” HIV services were issued following the initial stop‐work order, implementers faced many challenges in obtaining permission to resume HIV programming [11]. Early data from the Clinton Health Access Initiative from 14 African countries found disruptions in procurement of medications and decreases in oral PrEP initiations, HIV testing, paediatric case finding, early infant diagnosis and adult treatment [12]. These have resulted in substantial decreases in the delivery of HIV testing and treatment services for children and adults in African countries and are expected to compromise the gains made in preventing vertical HIV transmission, further exacerbating existing disparities in paediatric compared to adult HIV treatment [12, 13, 14].

The theme of the current supplement is how to optimize existing tools to end paediatric HIV and AIDS in the context of a rapidly changing funding environment. The included papers discuss how to maximize the impact of maternal and paediatric HIV prevention and treatment services, develop innovations in a time of reduced resources, and a framework to respond to the current crisis to improve maternal−child health services and put us back on the track to paediatric HIV/AIDS elimination.

Yakusik and colleagues review the drivers of vertical HIV transmission in sub‐Saharan Africa, noting important regional and national disparities [15]. In lower HIV prevalence western and central Africa, the primary determinant of vertical transmission is lack of maternal diagnosis and treatment access. In contrast, in high HIV prevalence eastern and southern Africa, the primary cause is new HIV acquisition by women during pregnancy or breastfeeding, accounting for 59% of vertical transmission in South Africa and 46% in Zambia. The authors estimate that preventing maternal HIV acquisition could reduce vertical transmission by approximately 25% in sub‐Saharan Africa, with the greatest effect in eastern and southern African countries. They then go on to model the impact and cost‐effectiveness of implementing targeted versus universal highly effective lenacapavir PrEP. Modelling demonstrated that geographically targeted lenacapavir PrEP implementation in high‐incidence settings would improve the PrEP cost‐effectiveness compared to a strategy of universal coverage. In sensitivity analyses, retention and service delivery costs were the primary drivers of cost‐effectiveness, with lower retention and higher service delivery costs substantially increasing net costs among all strategies. They note that any new PrEP intervention needs to be considered a complementary component to—rather than replacement for—core services for prevention of vertical transmission, address structural barriers, and be implemented alongside sustained investment in existing maternal and child health programmes.

Fowler and colleagues discuss the challenges of implementing current potent treatment regimens in women living with HIV (for both maternal health and preventing vertical transmission) in high‐burden countries in Africa, including structural factors affecting healthcare delivery, social and cultural issues, and the need for national commitment to adequately fund maternal and child health services [16]. Lessons learned from Botswana and Uganda in optimizing the perinatal HIV cascade are provided, with strong advocacy for innovative approaches to implement cost‐effective HIV care and treatment approaches for pregnant and breastfeeding women.

Chawana‐Mutingwende and colleagues present baseline data from a clinical trial conducted among peripartum adolescent girls and young women in the context of dolutegravir‐based ART and with concomitant peer support provided through the innovative Zvandiri community‐based model, delivering adherence support, psychosocial assistance and mental health counselling to young mothers [17]. Point‐of‐care viral load testing and urine tenofovir testing were done at baseline, as well as mental health evaluations. They found very high rates of viral suppression, with HIV RNA ≤40 copies/mL in 89% of this cohort, which were associated with detectable tenofovir levels and high self‐reported treatment adherence. Additionally, almost all participants reported no, minimal or mild post‐traumatic stress disorder, depression or anxiety. The Zvandiri model of peer support provides an innovative approach to optimizing care for high‐risk adolescent and young women during pregnancy and postpartum.

Important lessons from national programmes with success in preventing vertical HIV transmission are provided by Gaspar and colleagues in Brazil and Matshaba and colleagues in Botswana—countries that have been validated for certification by the World Health Organization (WHO) for maintaining vertical transmission rates <2% in non‐breastfeeding populations (Brazil) and <5% in breastfeeding populations (Botswana) [18, 19, 20]. In Brazil, success was attributed to a combination of universal health coverage, decentralized health programme governance, strong community engagement and integrated health information systems. In Botswana, the validation process was used to strengthen health systems and improve service delivery. Government ownership and leadership were viewed as critical, as was community engagement. Integrating HIV as part of a “triple elimination” strategy for HIV, syphilis and hepatitis B was used to strengthen all programmes concurrently. Both countries are now moving towards triple elimination goals [20].

The continuing disruptions in PEPFAR funding have led to concerns regarding the ability to achieve global vertical HIV elimination efforts [13, 14]. Siberry tackles how to reframe this financial and political upheaval into an opportunity to innovate and transform paediatric HIV prevention and care services into a framework that is less dependent on donor support [21]. He discusses the need to end HIV exceptionalism, with siloed HIV care, and fully integrate HIV services within a comprehensive maternal and child healthcare system aligned with national health services. National government leadership will be essential. Development of new partnerships between the public sector and pharmaceutical and private sectors to assist in serving population healthcare needs within a financially sustainable system that is less dependent on donor funding are also discussed. He notes the critical role of community needs and voices in the formulation of an evidence‐based, person‐centred, community‐informed, national health system‐aligned path to eliminating vertical HIV transmission in children and ensuring no child is left behind.

Another critical component to the elimination of paediatric HIV/AIDS is ensuring that care and treatment of children and adolescents who are living with HIV is optimized. Globally, more than 70,000 children living with HIV die annually due to AIDS‐related causes, despite clear evidence that very early initiation of ART—ideally in the first days of life—is critical to achieve the greatest clinical and immunologic benefit of therapy [9, 22, 23]. Thus, improving paediatric HIV case‐finding becomes a crucial factor in eliminating paediatric AIDS. Vojnov and Jani discuss the current gaps in paediatric HIV diagnosis and the importance of expanding case‐finding strategies beyond the traditional facility‐based early infant diagnosis testing [24]. Integration of paediatric HIV testing into healthcare systems to ensure linkage of infants across essential services and innovations in paediatric HIV testing with out‐of‐facility case‐finding approaches are discussed. The authors highlight mobile clinic and community‐testing, immunization clinic testing, family testing, index case‐finding and parental use of point‐of‐care HIV tests as potential models for countries to consider.

Very early initiation of ART is critical for the survival of infants living with HIV, but there has been a lack of potent antiretroviral drugs and formulations appropriate for infants, leaving less potent antiretroviral drug formulations having to be used for treatment in neonates [25]. Recent data from the PETITE‐dolutegravir clinical trial evaluating the pharmacokinetics and safety of dolutegravir in term neonates identified appropriate dosing of a scored 10 mg dolutegravir dispersible tablet over the first few weeks of life [26]. This trial also evaluated a novel, more easily administered 5 mg oral dispersible film formulation of dolutegravir, which is placed in the infant's mouth and disintegrates in saliva, rapidly dispersing systemically. The paper by Vijoen and colleagues describes the usability, safety and acceptability of the dolutegravir film formulation by mothers and healthcare workers, noting initial hesitancy followed by increased acceptability with use, and that targeted peer support was helpful [27]. Mothers and healthcare workers described the formulation as discreet, acceptable and easy to administer, once demonstrated to the mother. This new formulation offers a simple and acceptable way to provide the current potent standard of care, dolutegravir, to the youngest infants.

There are many challenges in the care of infants diagnosed with HIV, including fragmented service delivery, that have led to infants and young children having poorer outcomes across the HIV testing and treatment cascade than older children and adults. Tsiouris and colleagues advocate for a reimagining of paediatric HIV service delivery and discuss promising models of care that can improve outcomes and strengthen overall systems of care [28]. These include integrated mother−infant follow‐up models; family‐centred HIV care models treating the household as the unit of care; advanced HIV care strategies targeted to children with late presentation or treatment failure; and community‐based interventions including peer support, lay health workers and providing stigma‐free entry points to expand access and retention to paediatric (and maternal) care and treatment.

Finally, Bilinski and colleagues discuss the difficulties with trying to assess the impact of discontinuing US government funding assistance for reproductive, maternal and child health services in sub‐Saharan Africa on vertical HIV transmission) [29]. They note that services for prevention of vertical HIV transmission do not exist in isolation from other maternal and child health services. This becomes particularly evident when trying to assess the impact of interventions that need to reach women who are not living with HIV, such as lenacapavir PrEP. Optimal delivery of such interventions will require investments in family planning and antenatal and postnatal care platforms for mothers and children, which have been disrupted by the volatility and/or discontinuation of US government funding for such services in sub‐Saharan Africa.

As a whole, this supplement provides insights into the tools and innovations that can continue us on the path to ending paediatric HIV/AIDS in the context of a dramatically changing funding environment and ensure that children are not left (once again) behind. The papers also note the importance of care integration and provide further impetus towards achieving not just the elimination of vertical HIV acquisition but the triple elimination of HIV, syphilis and hepatitis B vertical transmission [20].

## Author Contributions

**Lynne M. Mofenson**: Writing – review and editing. **Jorge Pinto**: Writing – review and editing. **Angela Mushavi**: Writing – review and editing.

## Conflicts of Interest

LMM notes she received an honorarium for participation in a workshop panel in 2024 sponsored by Cepheid. JP and AM declare no conflicts of interest.

## Funding

The authors have nothing to report.

## Disclaimer

The authors alone are responsible for the views expressed in this issue. They do not necessarily represent the views, decisions or policies of the institutions with which they are affiliated, nor any of the funding agencies supporting their work.

## Data Availability

Data sharing is not applicable to this article as no datasets were generated or analysed during the current study.
